# Experimental Analysis of Bonding in Steel Glued into Pine Timber

**DOI:** 10.3390/ma17163897

**Published:** 2024-08-06

**Authors:** Adam Derkowski, Monika Chuda-Kowalska, Jakub Kawalerczyk, Dorota Dziurka, Radoslaw Mirski

**Affiliations:** 1 Department of Mechanical Wood Technology, Faculty of Wood Technology, Poznan University of Life Sciences, Wojska Polskiego 38/42, 60-627 Poznan, Poland; adam.derkowski@up.poznan.pl (A.D.); jakub.kawalerczyk@up.poznan.pl (J.K.); dorota.dziurka@up.poznan.pl (D.D.); 2 Institute of Structural Analysis, Faculty of Civil and Transport Engineering, Poznan University of Technology, pl. Sklodowskiej-Curie 5, 60-965 Poznan, Poland; monika.chuda-kowalska@put.poznan.pl

**Keywords:** reinforce timber, thermosetting adhesives, hybrid resin, strength of adhesive joints

## Abstract

Combining steel with wood has been practised for many years. The issue is related to two main areas, i.e., bonding steel elements with wood so that they serve as connectors facilitating the assembly of wood elements and bonding steel elements to wood beams to improve their load-bearing capacity. In the first case, the adhesives used may be relatively expensive and more difficult to apply, whereas in the second one, especially when steel elements are glued inside the glulam (GL) beams, it is better if the adhesives used are more accessible to apply and cheaper. As it seems rational to reinforce wood with high-modulus ties, research has been carried out to compare the connection quality of commercially available adhesives that can be used for this purpose. Moreover, thermosetting adhesives have been applied as an alternative and cheaper solution. Thermostat adhesives also have a high pH of the bond, which prevents the steel from rusting. The research shows that the load-bearing capacity of the bond depends on whether the bars are ribbed or sheet metal. Moreover, among thermosetting adhesives, the most favourable load-bearing values were obtained using a mixture of PF/pMDI (phenol formaldehyde resin/polymeric diphenylmethane diisocyanate) and powder from recycled tyres. The shear strength of these joints was 1.63 N/mm^2^ and 3.14 N/mm^2^ for flat specimens and specimens with ribbed bars, respectively.

## 1. Introduction

Construction is an industry with a substantial carbon footprint. Therefore, its growth implies the need for a more rational use of wood and a reduction in adverse environmental impacts. However, returning to wood in modern building structures is more complex. Along with its advantages, timber has many disadvantages, such as a lack of scalability, difficulties in sourcing long elements with the required load-bearing cross-sections, and a high dependence on load-bearing capacity on moisture content. Knots are a common cause of damage to both solid and glued timber beams. Knots and other defects are often removed and the resulting fragments are reconnected using a multilaminar joint [1,2,3,4,5]. However, the issue from a technical and procedural point of view has been sufficiently described in the standard guidelines [6]. Current work in this area analyses, among other things, the problems of making joints in wood with a moisture content above the fibre saturation point [7], joining hardwoods [8,9], or numerical modelling [10,11,12]. Another method that, in addition to compensating for the negative influence of knots, allows, at the same time, an increase in functional characteristics, such as an increase in load-bearing capacity or span of manufactured beams, is to reinforce GL beams with high-modulus tendons [7]. For practical reasons, composite materials based on carbon, glass, or basalt fibres, generally referred to as fibre reinforced polymers (FRPs), have found great acceptance. These materials, produced as mats, meshes, strips, or rods, are embedded in a polymer matrix in addition to the fibres above, which act as a kind of reinforcement. Epoxy, polyester, or vinyl ester resins are mainly used here. Among the most common polymer composites used for timber structures are materials based on carbon fibre (CFRP), basalt fibre (BFRP), glass fibre (GFRP) and aramid fibre (AFRP). The introduction of structures that cause the reinforcement of the tensile layer changes the position of the neutral axis in such a section.

On the one hand, this results in a more uniform operation of the distinguished zones of the beam, but on the other hand, in a so-called “changeover” situation, it can be a reason for beam failure in the compression zone [13,14,15,16]. The work related to reinforcing solid and glued timber beams is vast. It primarily concerns the effect of reinforcement on the stiffness of the beams or their load-bearing capacity [17,18,19], the impact of long-term loading [20,21,22], the species of timber reinforced in this way [23,24,25,26,27], or the renovation of the structure [28,29,30].

The work of Issa and Kmeid [31] indicated that strengthening beams with high-modulus materials (steel plate, CFRP strips) not only increases the load-carrying capacity of the beams but also changes the failure mode from brittle to ductile. Similar conclusions were drawn by De Luca and Marano [32]. In addition to indicating the beams’ failure mode, the authors showed an increase in the load-bearing capacity of about 40%. The degree of reinforcement was 0.82%. The adhesive used in this study was a two-component polyurethane resin (Purbond^®^ CR 421, Henkel & Cie. AG, Sempach Station, Switzerland).

In contrast, in the work of Xu et al. [33], using models describing the elastic-plastic behaviour of steel and the orthotropic elastic-plastic behaviour of wood, the numerical results coincided with experimental studies. The beams were analysed using 4-point bending, and the bars were glued into both zones using a two-component epoxy resin (RENOANTIC, Bulle, Switzerland). Of the eleven papers analysed from 1990 to 2021 on the assessment of the stiffness and static bending strength of steel tendon-reinforced beams, only in two cases were the bars glued in with single-component polyurethane [34,35], and the other nine were with different variants of epoxy resin [36,37,38,39,40,41,42,43,44]. However, all the publications analysed were concerned with high-strength adhesives that could be used at ambient temperatures (usually 20 ± 3 °C). They were therefore also suitable for use in the manufacturing of industrial beams. However, generally, in the publications analysed, the tendons were glued to existing beams and the joints were visible on the surface of the beams. However, beams were also analysed in which the tendons were glued in at the beam fabrication stage under the last layer of lumber in the tension zone [35,43]. Cold-bonding adhesives are justified because the layered glued beams are made in the cold, and tendons are most often carried out on beams already fabricated to increase their load-bearing capacity. The adhesives used are polyurethane and epoxy, which are expensive and are not widely used to manufacture GL beams. Melamine-based resins are much more commonly used. The glue joints formed by melamine-formaldehyde resin are considered among the most environmentally resistant and, therefore, ideal for glueing timber for wood structures. However, curing them at ambient temperature requires the use of very strongly acidic hardeners, which are, however, very damaging to steel, causing corrosion. Therefore, more information is needed in the literature on the possibility of using resins with a pH higher than 9.5 in steel-reinforced composite beams, which are relatively commonly used to glue wood together. This study’s main objective was to evaluate the load-bearing capacity of steel-wood glue joints made with thermosetting adhesives.

## 2. Materials and Methods

### 2.1. Materials

Pine wood (*Pinus sylvestris* L.), 0.5 mm thick steel plate (DC01), and ribbed steel bars with a diameter of 6 mm and class B500S (according to PN-H-93220:2006 [45], B500B according to DIN 488 [46] were used in the tests. The moisture content of the wood used was 8%, the bending strength was 119.6 N/mm^2^ (SD = 12.62 N/mm^2^), and the modulus of elasticity was 9855 N/mm^2^ (SD = 944 N/mm^2^). The properties of the pine wood were determined from a 3-point bending test on specimens with a cross-section of 20 × 20 mm and a length of 300 mm. The test was performed on 39 samples with an average density of 636 kg (SD = 52.5 kg).

**Figure 1:**
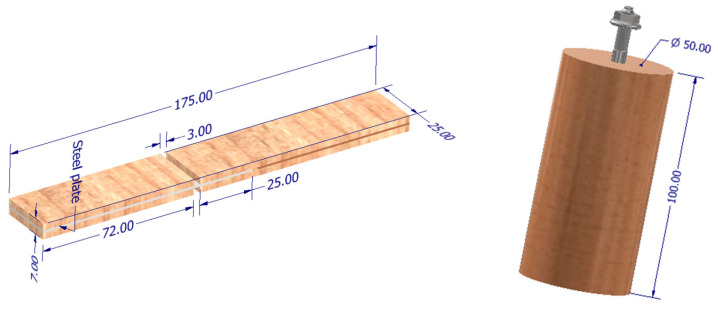
Illustrative drawing of the appearance of a sample used to assess the adhesion of adhesives to steel.

Cylindrical specimens with a diameter of 50 mm and a length of 100 mm and flat specimens were prepared from pine wood (Figure 1). A 6.1 mm diameter hole was drilled in the cylindrical specimens.

In addition, the following were used in the tests:

- A phenol-formaldehyde resin supplied by Silekol (Kędzierzyn-Koźle, Poland) with the marking R1010;
- A pMDI adhesive with the trade name Ongronat^®^ 2100 produced by BorsodChem Group (Kazincbarcika, Hungary).

In addition to the pure PF and pMDI adhesives, two glues were prepared with them:

- An adhesive with the marking PF70 containing 63.64% PF resin dry weight, 27.27% pMDI adhesive, and 9.09% rubber powder dry weight;
- An adhesive with the marking PF30 containing 27.27% PF resin dry weight, 63.64% pMDI adhesive, and 9.09% rubber powder dry weight.

Rubber granules resulting from the recycling of used tyres with a grain size of 0–0.4 mm, described by the manufacturer as powder, were used in the study. Under the trade name GREEN POWDER, this material came from Grupa Recykl (Śrem, Poland). The basic properties of adhesive compounds are presented in Table 1. All the agents used in the tests and presented in this table have properties similar to those commonly offered by the industry.

### 2.2. Preparation of Research Material–Methods

**Figure 2:**
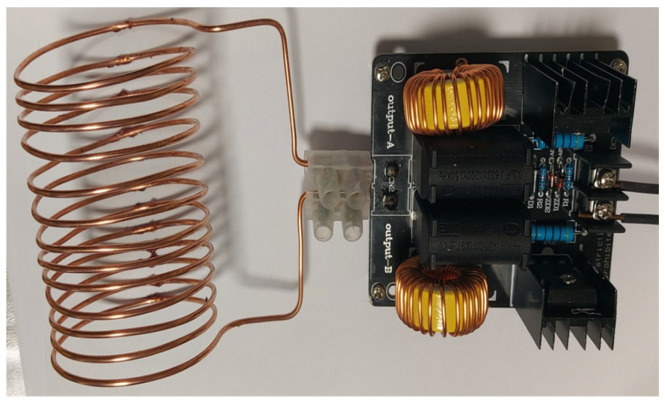
Induction heater used in the study.

The adhesives used in the study show high suitability for the production of wood materials and exhibit a high susceptibility to modifications, including mixing [47,48,49]. Similar curing temperature ranges further facilitate the process. Adhesives based on PF or pMDI resin mixtures must be cured at approximately 160–180 °C, which is also sufficient to re-vulcanize rubber from recycled tyres. Flat samples were decided to be cured at the adhesive bond curing temperature in a laboratory shelf press. The media shelves were heated to 180 ± 2 °C. The specimens were pressed at the unit pressure recommended for pressing soft species, i.e., 0.6 N/mm^2^ for 300 s. The adopted press time was based on the commonly accepted overheating time for wood mats when using phenol-formaldehyde resins, which is approximately 60 s per mm of press thickness. Cylindrical samples, on the other hand, were placed in a spiral made of copper wire. This helix was an induction coil with an inner diameter of 55 mm, a length of 100 mm, and 12 turns (Figure 2). The system was supplied indirectly from the mains, while it was provided directly via a stabilised DC voltage source with a continuously adjustable 0–30 V (DC). A dedicated system then generated a voltage at 85 kHz, which induced an inductive current flow in a steel element placed inside the coil. After initial tests, it was considered that the rods would be heated for 180 s by a heating system generating an average power of about 45 W.

The cured adhesives were also subjected to FTIR (Fourier-transform infrared) spectroscopy [50,51] and TG/TGA (thermogravimetric analysis/thermal gravimetric analysis) analysis [52,53]. Samples for testing were taken from the relevant adhesive joints immediately after the tests were performed. To prepare the test material, approximately 2 ± 0.01 g of the cured adhesives were ground in a laboratory grinder and sieved to obtain a 0.125 mm fraction. The powder, after drying, was mixed with KBr at a ratio of 1/200 mg. Therefore, 200 mg of the mixture was prepared and pressed into a unique steel ring under a pressure of 100 N/mm^2^, keeping the pressed mixture under vacuum to evacuate it. This yielded a transparent pellet fixed inside the ring, a holder during measurements. A Bruker FTIR IFS 66/s spectrometer (Bruker, Ettlingen, Germany) with a Fourier transform range of 500–4000 cm^−1^ was used for analysis, recording scans at a resolution of 4 cm^−1^. TG, DTA, and DSC (differential scanning calorimetry) analyses were also carried out for selected systems.

The tests were conducted using a Setaram’s LABSYS TM thermowell in open aluminium crucibles. Samples of cured resin weighing approximately 20 mg were heated at 5 °C/min in the temperature range of 20–600 °C in a helium atmosphere at a constant flow rate of 2 dm^3^/h.

**Figure 3:**
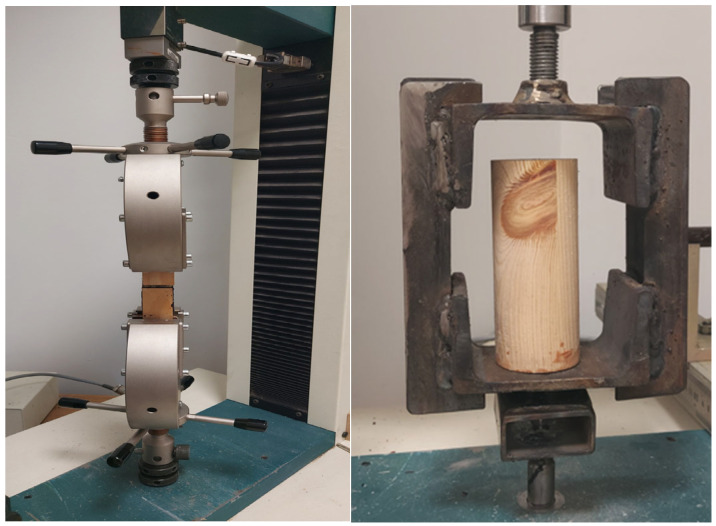
Photo of a flat and cylindrical sample placed in the holders of a testing machine.

Both flat and cylindrical specimens were subjected to a joint strength assessment against the wall in an axial tensile test. The strength assessment used a Tinus Olsen testing machine with a maximum load cell capacity of 10 kN. The samples were tested at a 0.5 mm/min crosshead feed rate. The position of the specimens in the holders of the strength machine is shown in Figure 3.

### 2.3. Date Analysis

Direct measurement results were gathered in an Excel spreadsheet. For each variant, 14 samples were prepared. The first verification of the obtained values was carried out. Using the Q-Dixon test [54], the set of results was checked for gross errors. Using the Q-Dixon test, two outliers were rejected. Excel 2019 was also used to prepare the data for statistical analysis. The other results obtained were statistically evaluated using the dedicated Statistica version 13.1 software. Due to the number of cases analysed, the results were subjected to an ANOVA [55] for assessment of variance. Post-hoc tests [56,57,58], i.e., Tukey’s test, were used to check the homogeneity of the groups. The results of these analyses are given in small letters in the graphs presenting the respective study. Generally, the mean and standard deviation (SD) were used in the study.

## 3. Results and Discussion

### 3.1. Effect of Adhesive Type of Load Capacity of the Connection

**Figure 4:**
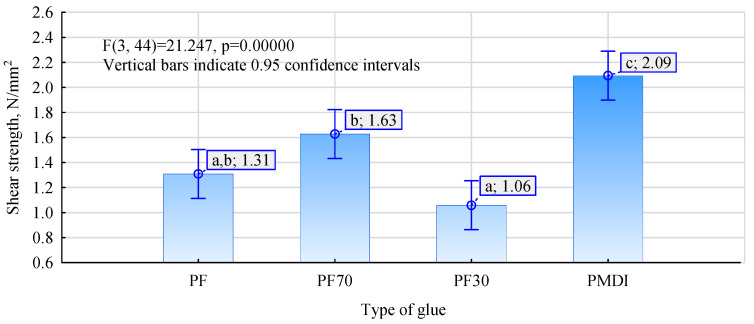
Effect of adhesive type on shear strength determined on flat specimens. Small letters mark homogenous groups.

Interesting results were obtained during tests to determine the possibility of using thermosetting resins to bond wood to steel on flat specimens. As can be seen from the values shown in Figure 4, the shear strength of the joint between the wood and the steel plate made with pMDI adhesive is significantly higher than in the other cases. A strength of 2.09 N/mm^2^ was obtained for this joint.

**Figure 5:**
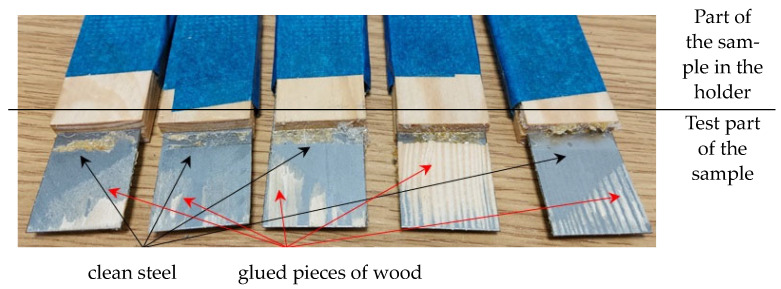
The surface of flat specimens after testing—jointly made on a pMDI basis.

This strength could likely be even higher, as significant areas with and without detached wood fibres could be found on the surface of the specimens, which were exceptionally clean—with no visible glue marks (Figure 5). This is probably because the surface of the steel plate was very smooth and shiny, showing neither traces of rust nor scratches, which minimised the adhesion of the glue to its surface.

**Figure 6:**
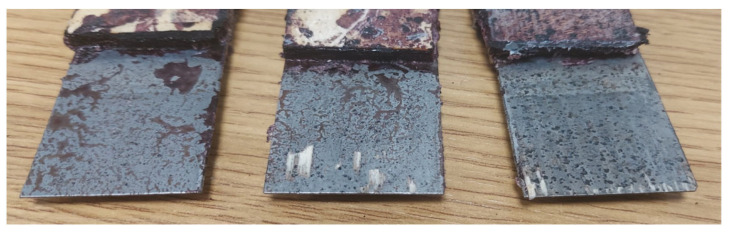
Surface of flat specimens after testing-joint was made with PF30.

For the other adhesive mixtures, significantly lower values were obtained. In addition, perhaps surprisingly, the worst result was obtained for a mixture containing about 70% pMDI adhesive dry matter and about 30% PF resin. In this case, the average strength was only 1.06 N/mm^2^. While there are no statistically significant differences between the average shear strength values determined for PF and PF30 systems, an adhesive mixture containing more pMDI should show better joint quality, as determined by shear strength. In contrast, a significantly higher, statistically similar value (there are no reasons to reject the null hypothesis of unequal averages) was shown by the adhesive mixture containing approximately 70% PF resin. The favourable change in reactivity of the PF resin, on the one hand, and, on the other, the marked change in viscosity of the system containing a significantly higher amount of water introduced into the pMDI with PF resin may be responsible for this. This fact was found organoleptically, but from the publication by Dziurka and Mirski [58], it appears that a mixture of UF resin (Urea-formaldehyde) with pMDI rapidly increases the dynamic viscosity, and the effect is more significant the more UF resin the mixture contained. This process occurs faster as PF resins have a much lower dry weight and, thus, a higher proportion of water. At this study stage, it is not easy to assess the effect of the ground tyre powder additive. Traces of it can be seen on the surface of the sheets after testing (Figure 6). It affects the texture of the prepared mixture, making it challenging to apply evenly to the smooth sheet surface. On the other hand, together with pMDI, swelling makes the cured joint very compact and rugged, improving the joint’s quality when using ribbed steel and eliminating defects in the grooves made for bar fitting.

**Figure 7:**
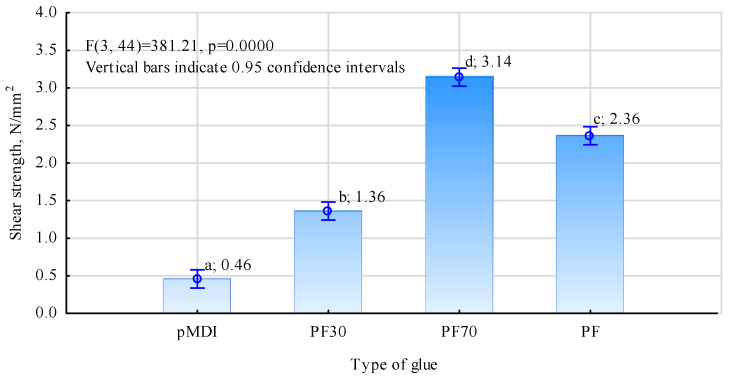
Effect of adhesive type on shear strength determined on cylindrical specimens. Small letters mark homogenous groups.

Similar efficiencies of the prepared adhesive mixtures were not obtained in tests performed on cylindrical specimens (Figure 7). Better results were obtained for systems with more PF resin. This difference may be due to two reasons:

- The ribbed rods do not adhere to the whole surface of the wood, and gaps are created between the wood surface and the rod core. This causes the adhesive to run down to the bottom of the groove where the rod is, with its initially lower viscosity;
- Isocyanate-based adhesives, although expanding, do not accept thick joints.

The cohesion of the free-curing pMDI adhesive is relatively low. This produces poor adhesive bond strength when free spaces exist between the rod core and the wood. The high-water content of the PF resin means that, although the resin seemingly increases in volume (becomes porous), the tested system tolerates joints of greater thickness better. Moreover, adding a filler in the form of rubber, which also swells during the curing process of the adhesive mixture, makes the joint more cohesive. As a result, voids are better filled, the glue joint has a less porous structure (which is the case with PF resin alone), and the mixture bonds the bars better to the surrounding wood. The observed strength increase of nearly 35% for the PF70 mixture is even more significant than that observed for the flat specimens. This may not be a legitimate comparison, as the shear strength values of the joint itself depend on many factors.

Nevertheless, the better adhesion for the PF70 mixture than pure PF resin in both variants is apparent. Although a sample with a markedly outlier shear strength of 6.1 N/mm^2^ was rejected, this result indicates the high potential of this system. Of course, at this stage of the research, it is challenging to determine the critical factor influencing the load-bearing capacity of the produced joints with pMDI/PF, as the quality of the joint may also depend on the maintenance of consistent relationships between the life of the mixture (reaction of the components at ambient temperature), the gelation time (crosslinking of the mixture at the process temperature), and the process temperature itself. In the case of flat specimens, the samples are subjected to a constant crosslinking temperature, as the ability to maintain the temperature of solid heating plates after heating is ±2 °C, and a few small specimens cannot affect even a momentary more significant change in their temperature. Induction heating, on the other hand, is subject to considerable error, although not enough to consider the process unsuitable for the tests performed.

It is difficult to find scientific papers on the strength of wood-steel joints describing the behaviour of thermosetting resins in this context. Most papers deal with various epoxy resin systems and occasionally with other resins, such as polyurethane (PUR) resins. Kemmsies [59] has shown that for different PUR resin systems, the shear strength takes on very different values, ranging from 2 N/mm^2^ to 8.65 N/mm^2^. The behaviour of PUR resin can be similar to pMDI. Parida et al. [60], following Aicher et al. [61], indicate that shear strengths in the range of 4.44 N/mm^2^ to 4.91 N/mm^2^ can be achieved using a two-component PUR resin. In this context, the results obtained are within the lower ranges of results obtained by other researchers. At this stage of the research, it is difficult to explain all the mechanisms involved, but it is known that compounds containing free isocyanate groups can form urethane bonds with metal [62,63]. The mixtures used, therefore, have potential but require further research work and in-depth analysis.

### 3.2. Thermogravimetric Analysis

**Figure 8:**
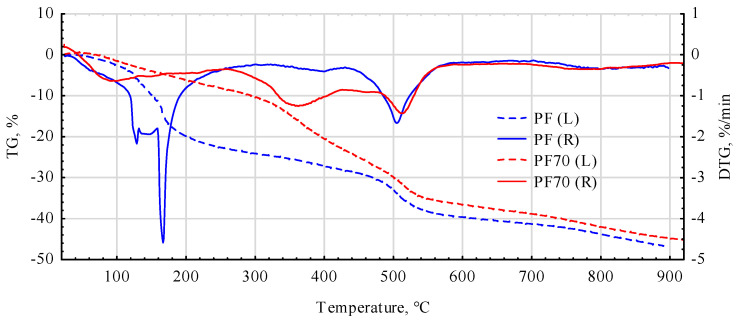
Thermograms of cured PF and PF70 (mix. PF, pMDI and rubber powder).

Analysis of the thermogravimetric TG and DTG curves indicates that the PF70 glue is much more thermally resistant than the pure PF resin (Figure 8). There are similarities for both bonding agents in the initial heating phase, i.e., in the temperature range of 40–120 °C and beyond about 540 °C. This area is generally considered to be the area where water and low-molecular-weight volatile crosslinking products evaporate. Significantly, the PF70 compound exhibits high heat resistance, as the weight loss is about 10%, compared to about 24% for pure PF resin.

**Figure 9:**
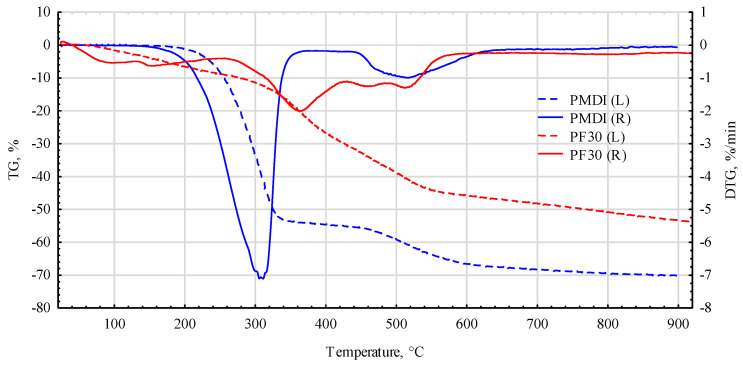
Thermograms of cured pMDI and PF30 (mix. PF, pMDI and rubber powder).

As can be seen from the data in Figure 9, the high thermal stability of the compounds used is mainly due to the tyre rubber derivatives [64] and pMDI. For this adhesive, a weight loss of about 10% occurs at a temperature of about 260 °C. The highest weight loss for pMDI, amounting to nearly another 45%, occurs in a very narrow temperature range, i.e., between 260 °C and 340 °C.

In the case of adding recycled tyre waste, a slight weight loss of about 10% only occurs when the system is heated to 360–370 °C. The prepared mixture, therefore, shows much more excellent stability in the range from 20 °C up to almost 580 °C. In its case, the weight loss is approximately 40 ± 5% and is only somewhat dependent on the PF/pMDI ratio. Thus, there is no need to worry about the substantial degradation of the used adhesive mixtures under induction heating.

As the thermal curves for the PF30 and PF70 mixtures differ only slightly, it was decided to present only one DTA curve in detail.

**Figure 10:**
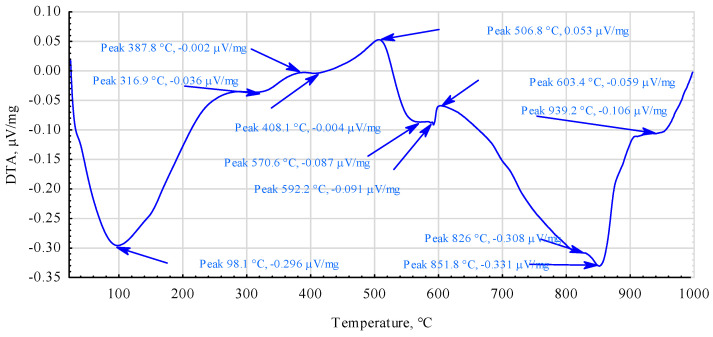
Thermograms of DTA of glue PF70.

Figure 10, therefore, shows a DTA curve for a mixture containing approximately 65% PF resin, 25% pMDI, and 10% recycled tyre powder. This curve indicates that, except in the temperature range of 400–520 °C, the thermal degradation of this mixture is exothermic.

From the data presented by Gou [65] and Da Silva [66], it appears that both PF resin and pMDI in the temperature range up to about 650 °C show an exothermic change course. At the same time, from the work of Yang and Roy [67], an endothermic peak is observed during the pyrolysis of the rubber in the temperature range of 450–500 °C, with a maximum peak at 465 °C. Therefore, the observed endothermic region, with a maximum peak at 506.8 °C in the analysed mixture (Figure 10), originates precisely from the decomposition of the rubber derivatives. The prepared mixture, therefore, appears to be sufficiently resistant to be thermally cured even by less stable heat sources.

### 3.3. Spectroscope Analysis

**Figure 11:**
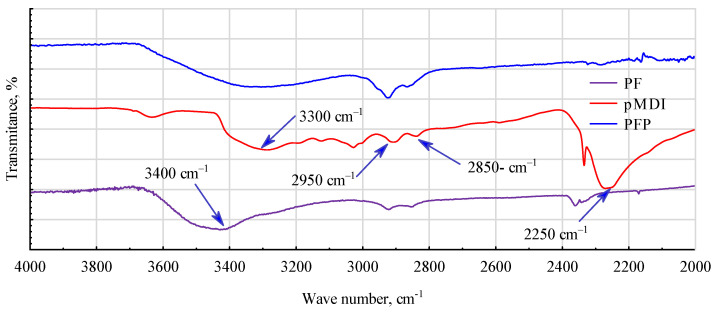
IR spectra of cured PF resin, pMDI adhesive and PF/pMDI mixture with added rubber powder (PFP).

The addition of rubber significantly affects the changes occurring in PF and pMDI resins. First, bands are disrupted between 2000 and 2800 cm^−1^ (Figure 11). The significant peak for pMDI at 2250 cm^−1^, which corresponds to the -NCO group, disappears. Only slight changes are observed in the bands at 2885 cm^−1^ or 2950 cm^−1^, attributed to stretching vibrations of the -CH and -CH2 bonds. The peaks attributed to O-H group vibrations at 3300 cm^−1^ and 3425 cm^−1^, for pMDI and PF, respectively, weaken.

**Figure 12:**
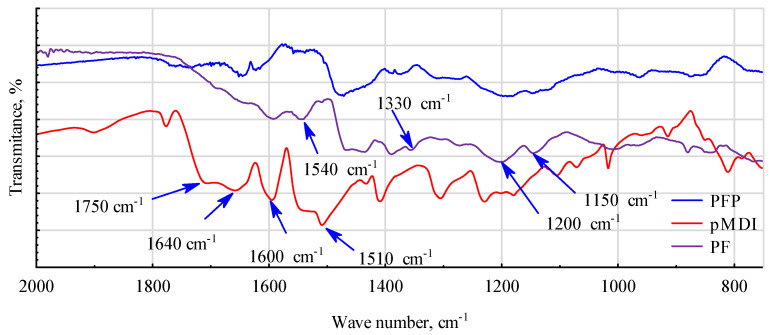
IR spectra of cured PF resin, pMDI adhesive and PF/pMDI mixture with added rubber powder (PFP)—range 800–2000 cm^−1^.

Typical peaks for pMDI at 1715 cm^−1^, 1600 cm^−1^, 1520 cm^−1^, and 1300 cm^−1^, associated with C=O, C=C, and C-H groups, and bands at 1150 cm^−1^ or 1200 cm^−1^, characteristic of PF resin, undergo some deformation and shift in modified PFP (Figure 12). The band at 1640–1650 cm^−1^, attributed to the amide groups of the urethane bonds (R-NH-C=O-R) for pMDI, and the bands at 1540 cm^−1^ and 1330–1390 cm^−1^, attributed in the PF resin to the stretching vibrations of the -CH bonds and the bending vibrations of the -OH bonds, are weakened. Thus, the changes observed in the FTIR spectra may be indicative of the excellent adhesion of pMDI to wood by reacting with the OH groups to form urethane bonds, and the excellent adhesion of the mixture used with steel allows the rods to remain in the wood [68,69]. This interpretation may be supported by the fact that the tests were carried out on resin samples taken from adhesive joints after destructive testing of samples.

## 4. Conclusions

The bonding of steel tendons to the timber structure, whether due to the desire to reinforce timber components or just as fasteners to facilitate assembly, can occur either after the preparation of the finished timber component or during its manufacturing. On the one hand, the choice of adhesive is determined by the need to maintain the load-bearing capacity of the resulting component and by application aspects.

Studies have shown that it is possible to use thermosetting adhesives used in the woodworking industry to join steel components with wood. An important aspect of thermosetting adhesives is providing sufficient heat energy for crosslinking the adhesive and ensuring adequate contact with the wood-glue-steel system.

When using ribbed steel, due to the free spaces between the wood and the rod core, it is necessary to use high-viscosity adhesives that do not flow by gravity during its crosslinking. A good solution in this case is to use a mixture of PF resin with pMDI, which, enriched with the addition of synthetic rubber, has a higher viscosity and increased reactivity than PF resin, which facilitates the application of the adhesive.

To ensure good contact between the materials to be bonded, and thus to ensure that a thin joint could be formed, the best results were probably obtained using pMDI as a bonding agent for wood and steel. The bond strength determined on flat specimens made with pMDI was as high as 2.09 N/mm^2^ and was nearly two times higher than the strength obtained with the PF30 mixture (the weakest joint).

The induction heating used in the study proved sufficient to cure the prepared adhesive mixtures. The thermal resistance, especially of the PF, pMDI, and tyre powder mixture, proved enough to ensure the durability of the bond. The best bond quality, as determined by shear strength, was obtained using a blend of 64% PF resin, 27% pMDI, and 9% recycled tyre powder (PF70). Its strength was 3.14 N/mm^2^.

Although we regard the research as preliminary, it appears that this type of solution may find practical applications. The PF or pMDI adhesives used in the study are widely available, and the rubber powder is additionally a recycled material. Thus, on the one hand, we have the possibility of increasing the load-bearing capacity of the wood and, on the other, the use of widely available adhesives and low-cost waste materials. Further studies should consider the quality of the preparation of the surfaces to be glued and the influence of environmental and ageing factors on the bond’s durability and the load’s variability. The final formulation and application technology should ensure both the durability and cost-effectiveness of the steel reinforcement wood with thermosetting adhesives.

## Figures and Tables

**Table 1:** Characteristic features of the adhesives used in the study.

Property	Unit	PF	pMDI	Rubber
Density	g/cm^3^	1.150	1.245	1.175
Dynamic viscosity	mPa·s	490	190	-
Dry matter content	%	49.5	100	99.2
pH	–	12.6	12.6	-
Gel time test at 130 °C	s	145	-	-
Hydrolytic chlorine	mg/kg	-	102	-
Content of NCO groups	%	-	30.3	-
Sulfur content	%	-	-	1 ÷ 3
Appearance	–	Clear, dark red liquid	Clear, dark brown liquid	Black powder

## Data Availability

The data are available on request from the correspondence author.

## Abbreviations

AFRP	Aramid fiber reinforced polymer
ANOVA	Statistic test. Analysis of variance
BFRP	Basalt fiber reinforced polymer
CFRP	Carbon fiber reinforced polymer
DC	Direct current
DSC	Differential scanning calorimetry
FRP	Fibre reinforced polymers
FTIR	Fourier-transform infrared spectroscopy
GFRP	Glass fiber reinforced polymer
GL	Glued laminated timber
PF	Phenol formaldehyde resin
PF30	Mixture of resin containing 27.27% PF resin dry weight, 63.64% pMDI adhesive, and 9.09% rubber powder dry weight.
PF70	Mixture of resin containing 63.64% PF resin dry weight, 27.27% pMDI adhesive, and 9.09% rubber powder dry weight
pH	pH scale indicates the activity of hydrogen ions in the solution
pMDI	Polymeric diphenylmethane diisocyanate
Post-hot	A group of statistical tests. They are used to uncover specific differences between three or more group means when an analysis of variance (ANOVA) test is significant
PUR	Polyurethane resin
Q-Dixona	Statistic test. The test is used for identification and rejection of outliers
SD	Standard deviation
TG	Thermogravimetric analysis
TGA	Thermal gravimetric analysis
Tukey’s test	Tukey’s test is a single-step multiple comparison procedure and statistical test.
UF	Urea-formaldehyde resin
